# Association between sleep duration and cardiovascular risk: the EVasCu cross-sectional study

**DOI:** 10.3389/fphys.2024.1430821

**Published:** 2024-07-26

**Authors:** Irene Martínez-García, Alicia Saz-Lara, Iván Cavero-Redondo, Iris Otero-Luis, María Dolores Gómez-Guijarro, Nerea Moreno-Herraiz, Samuel López-López, Carlos Pascual-Morena

**Affiliations:** ^1^ CarVasCare Research Group, Facultad Enfermería de Cuenca, Universidad de Castilla-La Mancha, Cuenca, Spain; ^2^ Facultad de Ciencias de la Salud, Universidad Autónoma de Chile, Talca, Chile; ^3^ Facultad de Enfermería de Albacete, Universidad de Castilla-La Mancha, Albacete, Spain; ^4^ Health and Social Research Center, Universidad de Castilla-La Mancha, Cuenca, Spain

**Keywords:** healthy adults, sleep duration, advanced glycation end products, intima-media thickness, pulse wave velocity, cardiovascular risk

## Abstract

**Introduction:**

Some cardiovascular risk markers have been associated with alterations in sleep duration in different populations; however, there is little evidence in a healthy population.

**Aim:**

The aim of the present study was to analyze the associations between sleep duration and cardiovascular risk biomarkers, including advanced glycation end-products (AGEs) measured by skin autofluorescence (SAF), maximum carotid intima-media thickness (IMT_Max_), aortic pulse wave velocity (a-PWV), pulse pressure (PP), and low-density lipoprotein cholesterol (LDL-C), in healthy adults (EVasCu study).

**Methodology:**

The EVasCu study included 390 participants. Simple and multiple linear regressions were performed between sleep duration and cardiovascular risk markers. ANOVA analysis and ANCOVA analysis adjusted for various covariates were then performed after categorizing sleep into 6 h, 6–8 h, and >8 h.

**Results:**

296 participants were included in the analyses (43.97 ± 12.60 years, 63.9% female). Simple linear regressions showed an inverse association between sleep duration and SAF, IMT_Max_, aPWV and PP. However, in the multiple linear regression with all the covariates, the statistical significance was lost. For its part, in the ANOVA analyses, sleep duration was also associated with the same parameters, but when performing the fully adjusted ANCOVA analyses, the statistical significance for SAF was maintained (*p* = 0.015), obtaining a difference of 0.223 arbitrary units (*p* = 0.017) when comparing the group <6 h vs. > 8 h. Finally, there was no association for LDL-C.

**Conclusion:**

An inverse association was found between sleep duration and APS, which is considered a marker of cardiovascular risk. Although prospective studies are needed, it is suggested that insufficient sleep may increase cardiovascular risk, which could be a key factor in future public health policies to promote health and prevent CVD.

## 1 Introduction

Sleep is recognized as a key behavioral factor in maintaining physical and mental health (Li et al., 2022). In recent years, changes in sleep patterns due to night shift work and 24/7 work demands have been observed, leading to subsequent sleep disturbances (Gohari et al., 2022). Additionally, age, habits and lifestyle, medical, psychological and/or medication conditions also affect to the circadian rhythms (Crowley, 2011; Onen and Onen, 2018; Dzierzewski et al., 2021). Recently, the highest prevalence of sleep disorders was reported in Europe following the COVID-19 pandemic (Linh et al., 2023); however, sleep duration varies between age groups and cultures, being generally longer in Europe (Olds et al., 2010; Gradisar et al., 2011). The gold standard for diagnosing sleep disorders is polysomnography (PSG) (Rundo and Downey, 2019), but actigraphy, a well-accepted and validated method for individuals with relatively adequate sleep patterns, is commonly used to measure sleep duration (Ancoli-Israel et al., 2003; Sadeh, 2011). Wearable devices, such as “activity wristbands,” are being used in current research because they are less expensive, more acceptable to the community, more convenient, less invasive, and can provide a better representation of usual sleep duration (Xie et al., 2018; Degroote et al., 2020).

The American Heart Association has included sleep as a component of the cardiovascular health score in its latest update “Life’s Essential 8” (Lloyd-Jones et al., 2022), highlighting the importance of studying the relationship between sleep and cardiovascular disease (CVD). Several biomarkers considered independent risk factors for CVD, including arterial stiffness, hypertension, hypercholesterolemia, atherosclerosis, and metabolic memory (or cumulative metabolic stress) (Lutgers et al., 2009; Yamagishi et al., 2015; Mancusi et al., 2018; Willeit et al., 2020; Valencia-Hernández et al., 2022), have previously been associated with sleep parameters (Gangwich et al., 2006; Nakazaki et al., 2012; Isami et al., 2018; Saz et al., 2022). Numerous observational studies have reported that reduced sleep duration or prolonged sleep is associated with the development of CVD and increased all-cause mortality (Cappuccio et al., 2011; Yin et al., 2017; Jike et al., 2018; Krittanawong et al., 2019; Krittanawong et al., 2020). However, other studies have failed to show a causal relationship between sleep duration and CVD (Liao et al., 2020; Zhuang et al., 2020); therefore, this relationship remains unclear.

Despite this apparent contradiction, the existence of a prior association between two variables may indicate a more complex or indirect relationship. Therefore, it is necessary to delve deeper into this association to understand the underlying mechanisms or intermediary factors that may influence cardiovascular health, thus improving our understanding of the relationship and allowing us to refute or validate previous findings. The main objectives of our study were to assess the association between sleep duration and cardiovascular risk measured by advanced glycation end products (AGEs) measured by skin autofluorescence (SAF), maximum carotid intima-media wall thickness (IMT_Max_), arterial pulse wave velocity (a-PWV), pulse pressure (PP), and low-density lipoprotein (LDL-Cholesterol, or LDL-C); and to compare the differences between the different cardiovascular risk variables and the different sleep duration groups, to identify sleep duration as a cardiovascular risk factor and to contribute to the knowledge of cardiovascular health.

## 2 Materials and methods

### 2.1 Study design and participants

This study was based on data from the cross-sectional EVasCu study, which was designed to validate a model of early vascular ageing (EVA) as an index of cardiovascular risk in healthy adults using confirmatory factor analysis (Saz et al., 2023). This study was approved by the Clinical Research Ethics Committee of the Cuenca Health Area (REG: 2022/PI 2022) and was conducted according to the guidelines for reporting observational studies, the Strengthening the Reporting of Observational Studies in Epidemiology (STROBE) Statement (Skrivankova et al., 2021), and in accordance with the guidelines and standards of the Declaration of Helsinki.

The sample size was calculated using Epidat software, which yielded a sample size of 355 participants, an estimated effect of 1, an alpha risk of 0.05, and an absolute precision level of 0.04 to detect a statistically significant result for the EVA index (Gomez et al., 2020).

Participants volunteered for the study after the project was disseminated through various media and social networks. Inclusion criteria were as follows: over 18 years of age, clinically stable for the previous 6 weeks, and provided written consent to participate in the study. Subjects enrolled in other studies with any type of intervention, receiving pharmacological treatment, or with a previous diagnosis of CVD (such as diabetes, hypertension, myocardial infarction, or stroke) were excluded from the study. The recruitment and measurement period lasted from June 2022 to December 2022 at the Health and Social Research Center of University of Castilla-La Mancha.

### 2.2 Sleep duration assessment

Sleep duration was assessed using the Xiaomi Mi Band activity wristband, a health and sports-focused wearable device that measures biomedical parameters including heart rate (HR), steps per day and sleep. The data from the wristband was obtained from the Zepp life application. The wristband was placed on the wrist of the non-dominant hand and data were collected for nine consecutive days; the first and last day of measurement were discarded to obtain the final average. For the remaining 7 days, the mean sleep duration in hours was estimated. Sleep duration was categorized according to the reviewed literature into three categories: “sleep duration less than 6 h,” “sleep duration between 6 and 8 h” and “sleep duration more than 8 h” (Cao et al., 2016; Niijima et al., 2016; Logan et al., 2018).

### 2.3 Skin autofluorescence evaluation

AGEs were measured over the SAF using the AGE Reader (DiagnOptics TechnologiesBV, Groningen, Netherlands). This device performs three consecutive individual measurements by autofluorescence, providing a more accurate and reliable final average of the measurements. Measurements were taken on both arms, avoiding skin imperfections such as scars, tattoos and birthmarks, and the resulting average was taken from both arms. Participants were asked not to use any skin creams prior to measurement to avoid interfering with the results (Noordzij et al., 2011). In addition, the light in the room was controlled, and a dark room was chosen for the measurements to avoid interference from any external light source. SAF values were expressed in arbitrary units (AU).

### 2.4 Maximum carotid intima-media wall thickness evaluation

Maximum IMT (IMT_Max_) was measured using the SonoSite MICROMAXX ultrasound device (SonoSite, Inc., Bothell, Washington, United States), a portable ultrasound system that allows assessment of carotid intima-media wall thickness. The patient was placed in the supine position, and the patient’s head was rotated to access the right and left carotid arteries. Both carotid arteries were located, and transverse and longitudinal images were obtained to clearly visualize the arterial wall. The thickness of the intima-media arterial wall was measured, and the maximum values of both carotid arteries were recorded. The average of the two values was then calculated (IMT_Max_ of the right carotid and IMT_Max_ of the left carotid). IMT_Max_ was estimated in millimeters (mm).

### 2.5 Arterial pulse wave velocity evaluation

The a-PWV was measured using a non-invasive oscillometric method, the Mobil-O-Graph^®^ (IEM GmbH, Stolberg, Germany), which provides values with acceptable accuracy compared to those of intra-aortic measurements (Hametner et al., 2013). Measurements were taken in a quiet place after a 5-min rest period using different cuffs depending on the participant’s arm circumference. The a-PWV was estimated in meters per second (m/s).

### 2.6 Pulse pressure evaluation

Two measurements, systolic blood pressure (SBP) and diastolic blood pressure (DBP), were taken using the OMRON^®^ M5-I blood pressure monitor (OMRON Healthcare UK Ltd.), with the cuff size corresponding to the participant’s arm circumference. These measurements were taken in a quiet place after a 5-min rest period and were separated by 5 min. The average of the two measurements was then taken to calculate the PP, which is the difference between SBP and DBP (PP = SBP-DBP). PP was estimated in milligrams of mercury (mm/Hg).

### 2.7 LDL-cholesterol

LDL-C was determined using the Roche Diagnostics^®^ Cobas 8,000 System. Blood samples were obtained between 8 and 9 a.m. after a 12-h fast. LDL-C was estimated in milligrams per deciliter (mg/dL).

### 2.8 Covariates

Demographic and clinical characteristics such as age, sex, educational level and smoking habits were collected by self-report questionnaires. Educational level was classified as follows: i) illiterate/no education; ii) primary education; iii) secondary education; iv) professional education/higher education; v) university education. Smoking habits were classified as follows: i) smoker; ii) ex-smoker <1 year; iii) ex-smoker 1–5 years; iv) ex-smoker >5 years; v) non-smoker. Family history (parents, children or siblings) of cardiovascular or cerebrovascular events was also asked. Skin phototype was estimated using the Fitzpatrick scale, which classifies skin color into six options, from the lightest (phototype I) to the darkest (phototype VI).

Glycated hemoglobin (HbA1c) was determined by high-performance liquid chromatography using the ADAMS A1c HA-8180V analyzer from A. Menarini Diagnostics^®^. Blood samples were collected by venous blood sampling between 8 and 9 a.m. after a 12-h fast.

To calculate body mass index (BMI), participants’ height was measured twice using a SECA 222 manual stadiometer (Seca^®^ GmbH and Co. KG, Hamburg, Germany), and the weight of the participants was measured using a scale SECA 869 (Seca^®^ GmbH and Co. KG, Hamburg, Germany), calculating the average of both measurements. BMI was calculated as the ratio of weight to height in square meters (kg/m^2^).

### 2.9 Statistical analysis

Normal probability plots and the Kolmogorov‒Smirnov test were used to test the normality of the distribution of continuous variables. Descriptive data for the total sample are presented as the means and standard deviations (SDs) or numbers of subjects and proportions (%), as appropriate.

A simple and multiple linear regression analysis was performed between the five cardiovascular risk biomarkers (SAF, IMT_Max_, a-PWV, PP and LDL-C) and sleep duration, using the Pearson statistic to analyze the correlation between the different variables and sleep duration. Model 1 was a simple linear regression between the different variables and sleep duration. Model 2 was a multiple linear regression adding sex and age to sleep duration. Finally, model 3 was performed, which differed according to the dependent variable. When the dependent variable was SAF, the independent variables were sleep duration, sex, age, educational level, smoking status, BMI, skin phototype and family history of acute myocardial infarction and/or stroke, while for the other dependent variables the independent variables were similar except for the exclusion of skin phototype and the inclusion of HbA1c. The exclusion of HbA1c as a covariable of the SAF is due to the close relationship between these markers. In the linear regressions, the slope (B), the statistical significance of the hours of sleep (*p*-value) and the correlation of the full model were considered using the Pearson r statistic.

In addition, an ANOVA analysis was performed, with *post hoc* analysis using the Bonferroni adjustment, using cardiovascular risk biomarkers as the dependent variable and sleep duration categorized as < 6 h, 6–8 h and >8 h as the independent variable. Two ANCOVA models were then performed, with *post hoc* analysis using the Bonferroni adjustment, adjusted for the same covariates as the models adjusted for multiple linear regression. In the ANOVAs and ANCOVAs, the differences between groups (B), the F-statistic and the *p*-values were considered.

Missing data were not included in the analyses. All statistical tests were 2-tailed, and the significance level was set at p0.05. IBM SPSS Statistics software (version 29.0; IBM Corp., Armonk, NY, United States) was used for data analysis.

## 3 Results

### 3.1 Characteristics of study participants

**FIGURE 1:**
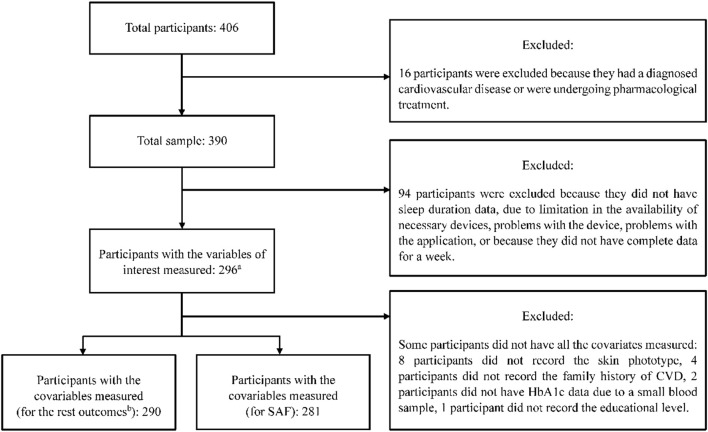
Flowchart of the study participants in the current study from the original EvasCu study. (a) For the SAF variable, 293 subjects had the measurement, in two participants, the measurement of SAF could not be carried out due to the dark color of their skin, and for one of the participants, due to tattoos on their arms. (b) For the IMT_Max_, a-PWV, PP and LDL-C.

Of the 390 study participants, sleep data was available for 296 participants. This reduction in the number of available participants was mainly due to software connection problems with the wristband. Of these 296 participants, 293 had data for the SAF, although this number was slightly reduced in the adjusted analyses when covariates were introduced (Figure 1). The mean age of the participants was 43.97 ± 12.60 years (63.9% female), the majority had a secondary education or higher, were non-smokers or ex-smokers, and had a normal BMI. When categorized by sleep duration, those who slept less than 6 h were more likely to be male (*p* = 0.004), and participants had elevated SAF (*p* < 0.001), IMT_Max_ (*p* = 0.022), a-PWV (*p* = 0.039) and PP (*p* = 0.018) (Table 1).

**TABLE 1:** Characteristics of the total sample and the different sleep duration categories.

Variables	Total (n = 296)	Categories for sleep duration, h			*p*-value
		≤6 h (n = 27)	6–8 h (n = 191)	≥8 h (n = 78)	
Age, years	43.97 (±12.60)	49.44 (±12.45)	43.83 (±11.96)	42.41 (±13.80)	0.042*
Sex, n (%)					
Males	107 (38.1%)	16 (59.3%)	72 (37.7%)	19 (24.4%)	0.004*
Females	189 (63.9%)	11 (40.7%)	119 (62.3%)	59 (75.6%)	
Education level, n (%)^a^					
Illiterate/no education	0 (0.0%)	0 (0.0%)	0 (0.0%)	0 (0.0%)	0.492
Primary education	3 (1.0%)	1 (3.7%)	1 (0.5%)	1 (1.3%)	
Secondary education	34 (11.5%)	4 (14.8%)	19 (9.9%)	11 (14.1%)	
Professional education/higher education	83 (28.0%)	5 (18.5%)	54 (28.3%)	24 (30.8%)	
University education	175 (59.1%)	17 (63.0%)	117 (61.3%)	41 (52.6%)	
Smokers, n (%)					
Smoker	32 (10.8)	4 (14.8%)	20 (10.5%)	8 (10.3%)	0.524
Ex-smoker <1 year	4 (1.4%)	0 (0.0%)	3 (1.6%)	1 (1.3%)	
Ex-smoker 1–5 years	11 (3.7%)	2 (7.4%)	7 (3.7%)	2 (2.6%)	
Ex-smoker > 5 years	57 (19.3%)	5 (18.5%)	43 (22.5%)	9 (11.5%)	
Nonsmoker	192 (64.9%)	16 (59.3%)	118 (61.8%)	58 (74.4%)	
BMI, n (%)					
Mean (±SD)	24.57 (±4.13)	26.02 (±4.30)	24.53 (±3.95)	24.14 (±4.44)	0.123
Under weight	10 (3.4%)	0 (0.0%)	5 (2.6%)	5 (6.4%)	
Normal weight	165 (55.7%)	11 (40.7%)	111 (58.1%)	43 (55.1%)	
Overweight	87 (29.4%)	9 (33.3%)	58 (30.4%)	20 (25.6%)	
Obesity	34 (11.5%)	7 (25.9%)	17 (8.9%)	10 (12.8%)	
Skin color, n (%)^b^					
FST I	28 (9.7%)	2 (7.7%)	18 (9.5%)	8 (10.9%)	0.113
FST II	129 (44.8%)	7 (26.9)	81 (42.9%)	41 (56.2%)	
FST III	84 (29.2%)	13 (50.0%)	59 (31,2%)	12 (16.4%)	
FST IV	40 (13.9%)	3 (11.5%)	26 (13,8%)	11 (15.1%)	
FST V	5 (1.7%)	1 (3.9%)	4 (2,1%)	—	
FST VI	2 (0.7%)	0 (0.0%)	1 (0,5%)	1 (1.4%)	
Family history of CVD, n (%)^c^					
Stroke and/or infraction	104 (35.6%)	12 (44.4%)	68 (36.0%)	24 (31.6%)	0.480
Without family history of CVD	188 (64.4%)	15 (55.6%)	121 (64.0%)	52 (68.4%)	
HbA1c, %^d^	5.21 (±0.33)	5.30 (±0.44)	5.20 (±0.31)	5.20 (±0.32)	0.324
Sleep duration, h	7.22 (±1.05)	5.17 (±0.62)	7.00 (±0.47)	8.50 (±0.56)	<0.001*
SAF, AU^e^	1.91 (±0.41)	2.19 (±0.57)	1.92 (±0.38)	1.80 (±0.38)	<0.001*
IMT_Max_, mm	0.45 (±0.13)	0.52 (±0.17)	0.45 (±0.13)	0.44 (±0.12)	0.022*
a-PWV, m/s	6.50 (±1.37)	7.09 (±1.48)	6.48 (±1.30)	6.32 (±1.42)	0.039*
PP, mmHg	45.94 (±10.63)	50.11 (±11.53)	46.29 (±9.66)	43.64 (±12.08)	0.018*
LDL-C, mg/dL	121.43 (±33.08)	125.89 (±33.06)	119.74 (±33.35)	124.00 (±33.50)	0.484

Continuous variables are expressed as the means ± standard deviations, and categorical variables are expressed as the number of subjects and percentages. HbA1c, Glycated hemoglobin; SAF, skin autofluorescence; AU, arbitrary units; IMT_Max_, Maximum thickness of the carotid intima-media wall; a-PWV, aortic pulse wave velocity; FST, fitzpatrick skin type; PP, pulse pressure; LDL-C, Low-Density Lipoprotein Cholesterol; CVD, cardiovascular disease.

^a^ The value of one of one subject was lost (Mean total value ± SD, for 295 subjects).

^b^ The value of eight subjects were lost (Mean total value ± SD, for 288 subjects).

^c^ The value of four subjects were lost (Mean total value ± SD, for 292 subjects).

^d^ The value of two subjects were lost in group of 6–8 h (Mean total value ± SD, for 294 subjects).

^e^ The value of three subjects were lost, two in group 6–8 h and one in group of ≥8 h (Mean total value ± SD, 293 subjects).

### 3.2 Association between sleep duration and cardiovascular risk biomarkers

Tables 2–4 show the results of the association between sleep duration and the different biomarkers of cardiovascular risk.

**TABLE 2:** Simple and multiple linear regressions for each of the cardiovascular risk variables and sleep duration.

Variable	Model 1			Model 2			Model 3		
	B	*p*-value	r	B	*p*-value	r	B	*p*-value	r
SAF	−0.077	0.001*	0.197	−0.045	0.023*	0.534	−0.038	0.060	0.549
IMT_Max_	−0.019	0.011*	0.147	−0.015	0.051	0.218	−0.015	0.055	0.245
a-PWV	−0.181	0.017*	0.139	0.021	0.497	0.919	0.020	0.498	0.924
PP	−1.161	0.049*	0.115	−0.396	0.458	0.474	−0.425	0.436	0.488
LDL-C	−0.161	0.930	0.005	2.008	0.242	0.413	2.511	0.140	0.486

Abbreviations: SAF, skin autofluorescence; IMT_Max_, maximum intima-media thickness; a-PWV, aortic pulse wave velocity; PP, pulse pressure; LDL-C, LDL-Cholesterol; B, slope; *r*, Pearson correlation. * indicates *p* < 0.05.

**TABLE 3:** ANOVAs for each of the cardiovascular risk variables and sleep duration categories.

Variable	Group			F, *p*-value	Post-hoc (Bonferroni)		
	<6 h	6–8 h	>8 h		<6 h vs. 6–8 h (B, *p*-value)	<6 h vs. > 8 h (B, *p*-value)	6–8 h vs. > 8 h (B, *p*-value)
SAF	2.185 ± 0.568 N = 27	1.921 ± 0.384 N = 189	1.799 ± 0.375 N = 77	F = 9.356 *p* < 0.001*	B = 0.264 *p* = 0.005*	B = 0.386 *p* < 0.001*	B = 0.122 *p* = 0.075
IMT_Max_	0.517 ± 0.172 N = 27	0.449 ± 0.131 N = 191	0.435 ± 0.124 N = 78	F = 3.876 *p* = 0.022*	B = 0.068 *p* = 0.043*	B = 0.082, *p* = 0.019*	B = 0.014 *p* = 1.000
a-PWV	7.093 ± 1.482 N = 27	6.484 ± 1.304 N = 191	6.319 ± 1.419 N = 78	F = 3.280 *p* = 0.039*	B = 0.609 *p* = 0.090	B = 0.773 *p* = 0.034*	B = 0.165 *p* = 1.000
PP	50.11 ± 11.53 N = 27	46.29 ± 9.66 N = 191	43.64 ± 12.08 N = 78	F = 4.090 *p* = 0.018*	B = 3.82 *p* = 0.236	B = 6.47 *p* = 0.019*	B = 2.65 *p* = 0.185
LDL-C	125.89 ± 30.06 N = 27	119.74 ± 33.35 N = 191	124.00 ± 33.50 N = 78	F = 0.728 *p* = 0.484	B = 6.15 *p* = 1.000	B = 1.89 *p* = 1.000	B = −4.26 *p* = 1.000

Abbreviations: SAF, skin autofluorescence; IMT_Max_, maximum intima-media thickness; a-PWV, aortic pulse wave velocity; PP, pulse pressure; LDL-C, LDL-Cholesterol; B, difference between groups; F, F value of ANOVAs/ANCOVAs. * indicates *p* < 0.05.

**TABLE 4:** ANCOVAs for each of the cardiovascular risk variables and sleep duration categories.

Variable	Model 1				Model 2			
	Overall	*Post-hoc* (Bonferroni)			Overall	*Post-hoc* (Bonferroni)		
	F, *p*-value	<6 h vs. 6–8 h (B, *p*-value)	<6 h vs. >8 h (B, *p*-value)	6–8 h vs. > 8 h (B, *p*-value)	F, *p*-value	<6 h vs. 6–8 h (B, *p*-value)	<6 h vs. > 8 h (B, *p*-value)	6–8 h vs. > 8 h (B, *p*-value)
SAF	F = 5.779 *p* = 0.003*	B = 0.174 *p* = 0.052	B = 0.269 *p* = 0.003*	B = 0.095 *p* = 0.141	F = 4.267 *p* = 0.015*	B = 0.122 *p* = 0.267	B = 0.223 *p* = 0.017*	B = 0.101 *p* = 0.112
IMT_Max_	F = 2.238 *p* = 0.108	B = 0.054 *p* = 0.151	B = 0.063 *p* = 0.122	B = 0.008 *p* = 1.000	F = 1.929 *p* = 0.147	B = 0.053 *p* = 0.188	B = 0.058 *p* = 0.189	B = 0.005 *p* = 1.000
a-PWV	F = 0.063 *p* = 0.939	B = −0.019 *p* = 1.000	B = −0.040 *p* = 1.000	B = −0.021 *p* = 1.000	F = 0.064 *p* = 0.938	B = −0.039 *p* = 1.000	B = −0.029 *p* = 1.000	B = 0.010 *p* = 1.000
PP	F = 0.792 *p* = 0.454	B = 1.28 *p* = 1.000	B = 2.49 *p* = 0.748	B = 1.22 *p* = 1.000)	F = 0.970 *p* = 0.382	B = 1.07 *p* = 1.000	B = 2.61 *p* = 0.709	B = 1.55 *p* = 0.720
LDL-C	F = 1.205 *p* = 0.301	B = −0.62 *p* = 1.000	B = −6.83 *p* = 0.983	B = −6.21 *p* = 0.394	F = 1.485 *p* = 0.228	B = −2.49 *p* = 1.000	B = −8.99 *p* = 0.576	B = −6.49 *p* = 0.339

Abbreviations: SAF, skin autofluorescence; IMT_Max_, maximum intima-media thickness; a-PWV, aortic pulse wave velocity; PP, pulse pressure; LDL-C, LDL-Cholesterol; B, difference between groups; F, F value of ANOVAs/ANCOVAs; * indicates *p* < 0.05.

Simple linear regressions (model 1) showed a strong inverse association between sleep duration and cardiovascular risk biomarkers, except for LDL-C. Sleep duration was associated with SAF (B = −0.077, *p* = 0.001), IMT_Max_ (B = −0.019, *p* = 0.011), a-PWV (B = −0.181, *p* = 0.017) and PP (B = −1.161, *p* = 0.049). After adjustment for age and sex, the association remained statistically significant only for SAF (B = −0.045, *p* = 0.023) and maintained a trend towards significance for IMT_Max_ (B = −0.015, *p* = 0.051). Finally, in the fully adjusted model (model 3), statistical significance was lost for all cardiovascular risk biomarkers, although a trend remained for SAF (B = −0.038, *p* = 0.060) and IMT_Max_ (B = −0.015, *p* = 0.055).

ANOVA analyses revealed statistically significant associations between categorized sleep duration and the different cardiovascular risk biomarkers, except for LDL-C (F = 9.356 and *p* < 0.001 for SAF, F = 3.876 and *p* = 0.022 for IMT_Max_, F = 3.280 and *p* = 0.039 for a-PWV, and F = 4.090 and *p* = 0.018 for PP). Bonferroni *post hoc* adjustment showed that for the comparison <6 h vs. 6–8 h, SAF was increased by 0.264 AU (*p* = 0.005) and IMT_Max_ by 0.068 mm (*p* = 0. 043), whereas for the comparison <6 h vs. > 8 h, SAF increased by 0.386 AU (*p* < 0.001), IMT_Max_ by 0.082 mm (*p* = 0.019), a-PWV by 0.773 m/s (*p* = 0.034) and PP at 6.47 mmHg (*p* = 0.019). In the ANCOVA analyses, statistical significance was maintained only for the SAF (F = 5.779 and *p* = 0.003 for model 1, and F = 4.267 and *p* = 0.015 for model 2). Thus, with Bonferroni adjustment, <6 h vs. > 8 h increased SAF by 0.269 AU (*p* = 0.003) and 0.223 AU (*p* = 0.017) in models 1 and 2, respectively.

## 4 Discussion

Our study showed that in simple linear regressions and ANOVAs, sleep duration is strongly associated with cardiovascular risk biomarkers, i.e., SAF, IMT_Max_, a-PWV and PP, with a deleterious association, i.e., lack of sleep increases these biomarkers. When age and sex were included in the analyses, most of the associations were lost, except for the association between hours of sleep and SAF, a pattern that was maintained in the fully adjusted model in the ANCOVA. However, an association for IMT_Max_ cannot be excluded, as there was a trend in the multiple linear regressions. Finally, there was no association for LDL-C in any of the analyses.

Our results show that sleep duration has an inverse and statistically significant relationship with SAF. Specifically, multiple linear regression showed a non-statistically significant trend towards a reduction of 0.038 AU per hour slept. This result is likely to be higher, as shown by the fully adjusted ANCOVA, in which subjects who slept less than 6 h had an average of 0.223 AU more than those who slept more than 8 h. These results are consistent with previous reports (Nomoto et al., 2012; Isami et al., 2018). Specifically, one of these studies obtained a result very similar to ours, with a regression coefficient of −0.030, which was statistically significant (Isami et al., 2018). Other previous studies in populations with chronic obstructive pulmonary disease have found interesting associations between sleep and AGEs. Apnea severity and sleep duration were associated with AGEs, independent of other variables that could influence them (age, BMI, smoking, or basal plasma glucose). Although our study is cross-sectional and causality cannot be inferred, it is somewhat likely given the previous literature and some mechanisms that could explain it. Thus, AGEs are reduced after treatment with continuous positive airway pressure, which improves the quality and quantity of sleep, supporting the idea of a possible causality in the association found in our study (Tan et al., 2006; Lam et al., 2012; Wu et al., 2018). There are some mechanisms that can be derived from the alteration of sleep parameters, such as metabolic changes, activation of inflammatory pathways or increased sympathetic nervous system activity (Nilsson et al., 2001; Meier-Ewert et al., 2004; Schmid and Schultes, 2011), causing higher oxidative stress, which could stimulate the formation and accumulation of AGEs (Isami et al., 2018).

Although it was decided to exclude HbA1c from the adjusted analyses because of its close relationship with SAF/AGEs, preliminary analyses showed no effect on the adjusted analyses when this covariate was introduced. Thus, bivariate correlations showed a strong association between age and SAF (B = 0.018, r = 0.560, *p* < 0.001), and a slightly lower correlation between age and HbA1c (B = 0.010, r = 0.393, *p* < 0.001) and between HbA1c and SAF (B = 0.387, r = 0.310, *p* < 0.001). However, when multiple linear regression was performed with SAF as the dependent variable and age and HbA1c as independent variables, age remained similar (B = 0.016, *p* < 0.001), while the association with HbA1c decreased significantly (B = 0.126, *p* = 0.030). When the remaining covariates, including HbA1c, were introduced to obtain the fully adjusted model, HbA1c lost statistical significance (*p* = 0.166). These results are partially consistent with a previous study (Turk et al., 1998). In this study, conducted in a population with diabetes, a strong correlation was observed between HbA1c and AGEs in HbA1c (r = 0.77). Furthermore, according to glycemic control estimated by HbA1c, the correlation was higher in participants with poorer glycemic control (r = 0.51) compared to participants with better glycemic control (r = 0.37). These values are consistent with our results because, although we did not include participants with diagnosed diabetes, the correlation was comparable to that of participants with well-controlled diabetes. However, in our case, age was very strongly associated with SAF, unlike in that study, where age was not associated with AGEs in HbA1c. This may be because SAF estimates AGEs in the skin, which accumulate over long periods of time, partly independently of glycemic control, and are more related to tissue-level glycation due to unhealthy lifestyles, including (but not exclusively) glycemic control. While AGEs in HbA1c have their own biosynthesis, pharmacokinetics and clinical significance, they are different (although related) to AGEs in skin. Finally, although already mentioned, it should not be overlooked that our population is a priori healthy, where HbA1c and therefore AGEs in HbA1c are expected to be at normal levels, whereas SAF is not a direct indicator of disease, so participants could have elevated SAF without apparent disease.

Sleep duration was associated with all cardiovascular risk markers in unadjusted models; however, significance was lost in all of them after adjustment. This lack of statistical significance could be due to several factors. First, there may be covariates that modify the association obtained and of which we are unaware, although we have carried out a prior study of the variables that had to be included in the adjusted models. In addition, age and sex are associated with cardiovascular risk biomarkers, and age is also associated with sleep duration, which means that when these variables are introduced into the models, some or all the statistical significance is lost, leaving only the association between SAF and sleep duration. This may indicate that age and/or sex are confounding variables for some outcomes, such as a-PWV or PP. Specifically, the correlation between age and a-PWV is almost perfect (*r* = 0.918, *p* < 0.001), with differences also observed between sexes, though with less statistical significance (*p* = 0.037); for IMT_Max_, the correlation with age is lower (*r* = 0.118, *p* = 0.020), but with sex it was higher (*p* = 0.006). The correlation between age and PP was not statistically significant, although there was a clear trend (r = 0.088, *p* = 0.084), while there was a high statistical significance according for sex (*p* < 0.001). LDL-C was not associated with sex (*p* = 0.700) but was highly correlated with age (*r* = 0.442, *p* < 0.001). Finally, age was highly associated with SAF (*r* = 0.560, *p* < 0.001), but not with sex (*p* = 0.489). This could be the reason why, in our sample, the introduction of age and sex lost most of the statistical significance in adjusted analyses, i.e., the stronger the association of age and/or sex with the dependent variables, the greater the likelihood that the association would be missing in adjusted models, especially considering that age and sex are also moderately associated with sleep duration (*r* = 0.152 and *p* = 0.009, and *p* = 0.019, respectively). It should be noted that in future studies with larger sample sizes that allow for robust results to be obtained, interaction analyses by sex and age will also be carried out. However, as the multiple linear regression for IMT_Max_ tended towards statistical significance (*p* = 0.055), it is likely that an association would be observed with a larger sample size. This would be consistent with previous reports in which short sleep duration was associated with increased IMT (Wolff et al., 2008; Nakazaki et al., 2012; Sands et al., 2012).

The inverse association between SAF and sleep duration, and probably also IMT_Max_, is of great interest at a population level. Although this study cannot prove causality, it is reasonable to hypothesize that the cause of the increase in SAF, and perhaps IMT_Max_, may be due to a lack of sleep. If this is proven, short sleep duration could increase cardiovascular and other morbidity and mortality. In any case, our study is consistent with other authors. Thus, previous reports showed that sleeping less than 6 h is associated with increased cardiovascular risk and mortality (Itani et al., 2017; Li et al., 2022). Krittanawong et al. (2019) reported that this increased risk occurred with a sleep duration of less than 7 h. On the other hand, Nakazaki et al. (2012) conducted a study in an elderly population and reported that cardiovascular risk increased when sleep duration was less than or equal to 5 h. This finding was also reported in a study by Gangwich et al. (2006), who reported an increased risk of hypertension associated with sleeping less than or equal to 5 h per night in adults aged 32–59 years. These findings suggest that age is a factor to consider when recommending sleep duration, as risk varies with this variable. Currently, CVD represents a major challenge due to its high incidence and prevalence, with crude CVD mortality increasing in both sexes in several European countries (Timmis et al., 2020). Short sleep duration could therefore be a key factor in reducing mortality and CVD incidence and should be addressed in clinical practice through psychoeducation or psychosocial intervention (Itani et al., 2017).

However, in this study, no significant increases in the variables of interest were found in the group with more than 8 h of sleep, as noted in an umbrella review that showed a U-shaped relationship between sleep duration and cardiovascular risk (Li et al., 2022). This may be due to the nature of the population studied, as this relationship has previously been studied in populations with preexisting pathologies, while this is one of the first studies to estimate this association in a healthy population. It may also be due to the fact that recommendations for sleep duration differ between age groups, with non-recommended durations being more than 9 h of sleep in those over 65 years of age and more than 10 h in adults between 26 and 64 years of age, according to the National Sleep Foundation (Hirshkowitz et al., 2015). In our study, the age ranges used in previous studies (Cao et al., 2016; Niijima et al., 2016; Logan et al., 2018) were chosen to classify long duration sleep as 8 h or more per night to facilitate subsequent comparisons.

This study, conducted in a healthy population, may have some implications. First, prospective studies evaluating sleep duration and cardiovascular risk biomarkers, particularly SAF and IMT_Max_, are needed to establish causality with greater certainty. In addition, it would be of great interest to investigate the specific pathophysiological mechanisms by which sleep deprivation increases SAF. Second, assuming causality is established, public health policy should emphasize sleep duration and quality as protective factors against cardiovascular events. Although the individual impact would necessarily be small, significant reductions in morbidity and mortality could be achieved at a population level. Third, the use of wristbands by the population may be of interest for individual monitoring of health status and lifestyle, such as physical activity, cardiorespiratory status and sleep.

Our study has several limitations, the most important of which is its cross-sectional design, which prevents causal inference. Therefore, our results cannot be generalized to other populations. In addition, most of the participants were Caucasian. Our study did not assess sleep regularity with consistent schedules, time of waking or going to bed, or sleep quality, factors that are also associated with adverse health outcomes (Chaput et al., 2020). Napping has been associated with an increased risk of cardiovascular events in those who sleep more than 6 h (Wang et al., 2019), but this was not assessed in our study. The effect of the weekend on daily sleep was also not assessed, but previous studies have reported that it usually compensates for any sleep deficit during the week (Roepke and Duffy, 2010). Another limitation could be the device used to measure sleep duration, as a previous study showed an overestimation of sleep compared to a validated actigraphy when using an earlier model of activity tracker (Xiaomi Mi Band 2) (Kubala et al., 2020). However, another study assessed the validity of several wearable devices and found them to be highly accurate for measuring sleep duration (Xie et al., 2018). The small sample size, as well as some missing data, is another major limitation affecting the reliability, validity, and applicability of the results. Finally, despite the inclusion of covariates that were considered relevant to this study and its objective, there may be some that were not included, especially if they were not measured in our study (e.g., sleep quality).

In conclusion, cardiovascular risk variables, except LDL-C, were inversely associated with sleep duration in the healthy population in both linear regressions and ANOVAs. This association was maintained in adjusted models, particularly in the association between sleep duration and SAF in the ANCOVA analyses, with participants with higher SAF being those who slept less than 6 h. These findings may explain part of the pathophysiology of CVD progression, and additional studies are needed to confirm this possible causal relationship. The findings also highlight the importance of sleep for population health, which could be a key factor in future public health policies to promote health and prevent CVD.

## Data Availability

The raw data supporting the conclusions of this article will be made available by the authors, without undue reservation.
